# Identification of genomic features in the classification of loss- and gain-of-function mutation

**DOI:** 10.1186/1472-6947-15-S1-S6

**Published:** 2015-05-20

**Authors:** Seunghwan Jung, Sejoon Lee, Sangwoo Kim, Hojung Nam

**Affiliations:** 1 School of Information and Communication Department, Gwangju Institute of Science and Technology, 123 Cheomdangwagi-ro, Buk-gu, Gwangju, Republic of Korea; 2 Bio-Synergy Research Center, Daejeon, Republic of Korea; 3 Severance Biomedical Science Institute, Yonsei University College of Medicine, Seoul 120-752, Korea

## Abstract

**Background:**

Alterations of a genome can lead to changes in protein functions. Through these genetic mutations, a protein can lose its native function (loss-of-function, LoF), or it can confer a new function (gain-of-function, GoF). However, when a mutation occurs, it is difficult to determine whether it will result in a LoF or a GoF. Therefore, in this paper, we propose a study that analyzes the genomic features of LoF and GoF instances to find features that can be used to classify LoF and GoF mutations.

**Methods:**

In order to collect experimentally verified LoF and GoF mutational information, we obtained 816 LoF mutations and 474 GoF mutations from a literature text-mining process. Next, with data-preprocessing steps, 258 LoF and 129 GoF mutations remained for a further analysis. We analyzed the properties of these LoF and GoF mutations. Among the properties, we selected features which show different tendencies between the two groups and implemented classifications using support vector machine, random forest, and linear logistic regression methods to confirm whether or not these features can identify LoF and GoF mutations.

**Results:**

We analyzed the properties of the LoF and GoF mutations and identified six features which have discriminative power between LoF and GoF conditions: the reference allele, the substituted allele, mutation type, mutation impact, subcellular location, and protein domain. When using the six selected features with the random forest, support vector machine, and linear logistic regression classifiers, the result showed accuracy levels of 72.23%, 71.28%, and 70.19%, respectively.

**Conclusions:**

We analyzed LoF and GoF mutations and selected several properties which were different between the two classes. By implementing classifications with the selected features, it is demonstrated that the selected features have good discriminative power.

## Background

A mutation refers to a change of the genomic sequence, which contains all of the genetic information of an organism. Because proteins are generated and regulated based on the genome sequence, alterations of the genome can lead to changes of protein functions [1]. Through these genetic mutations, a protein can loss its native function (loss-of-function), or it can confer a new function (gain-of-function) [2-5]. For example, a mutated fumarate hydratase (FH) loses its native catalytic activity [6], and heterozygous point mutations in isocitrate dehydrogenase (IDH1, IDH2) confer a new metabolic enzymatic activity that produces 2-hydroxyglutarate [7,8]. In addition, in the FGFR1 gene, GoF and LoF mutations can lead to different diseases, craniosynostosis and Kallmann syndrome, respectively [9-12]. Therefore, it is important to understand the characteristics of functional mutations and to determine which mutations lead to LoF and GoF results for clinical target.

There are many studies of mutations, including LoF and GoF mutations. MacArthur *et al*. implemented a systematic survey of LoF variants. They showed many LoF variant properties compared to other mutations, such as the allele frequency and the degrees of associations with diseases. They also showed the effects of LoF variants, including phenotypes, diseases, and gene expressions. However, missense mutations were excluded from the LoF mutations which they defined [4]. In our study, many mutations were missense mutations; therefore, we chose to address missense mutations. Reva *et al*. estimated functional effects of missense mutations using evolutionary conservation information [13]. Lee *et al*. discussed the bi- directional SIFT (B-SIFT), which is a modified form of SIFT. In addition, the B-SIFT algorithm calculates scores of mutation alleles based on evolutionary conservation information [3]. They used the scores to identify mutations which cause hyperactivation or gain-of-function outcomes, but our work uses not only the functional effects of mutations but also several other properties. However, most previous studies focused on either LoF or GoF mutations or on functional changes in a specific gene.

**Figure 1:**
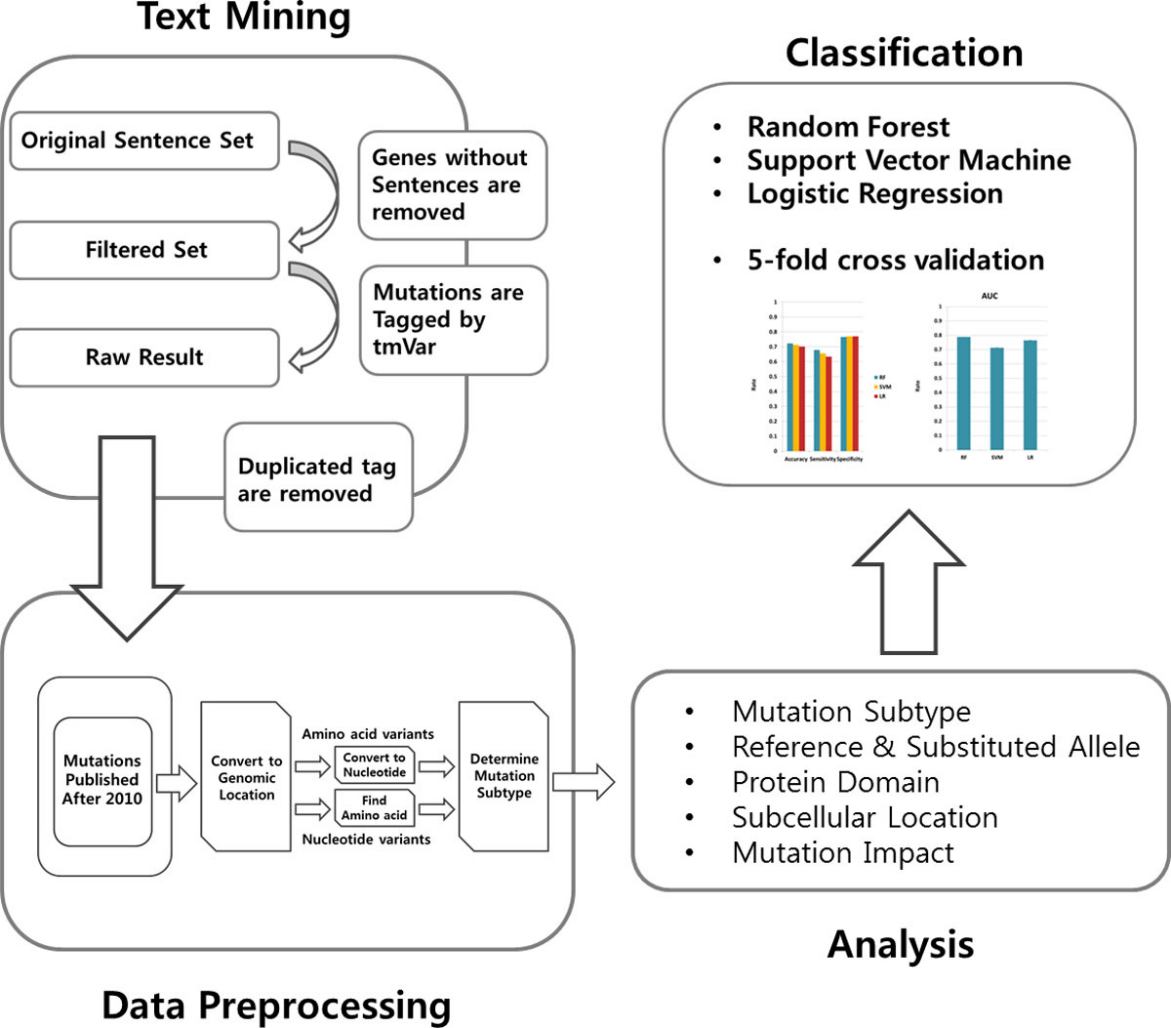
**The flow chart of this study**. An overview of the analysis of LoF and GoF features is given. First, from the literature, we obtained LoF and GoF mutation information. Next, we applied a data-preprocessing step. During this step, we converted amino acid locations into genomic locations and amino acid residues into 3-mer nucleotide base alleles, after which we determined mutation subtypes. In addition, mutations whose reference allele was not matched to a reference genome or mutations published before 2009 were removed. Next, with the remaining mutations, features which have discriminative power between LoF and GoF mutations were extracted. Lastly, three classifications were implemented using the selected features to confirm whether the features can be used to classify the two classes.

In this work, we propose a comprehensive analysis of the genomic features in mutations to classify LoF and GoF mutations. Figure 1 shows an overview of our study. First, from the literature, 14,259 gene-sentence relations for GoF and 29,586 relations for LoF were determined. By removing genes without sentences and extracting mutations and their locations from the sentences, we obtained information on 816 LoF and 474 GoF mutations. Next, we applied a data-preprocessing technique. During this process, mutations with an amino acid location were converted into those with a genomic location, and amino acid residues were converted into 3-mer nucleotide alleles, after which the mutation subtypes were determined. In addition, mutations whose reference allele is not matched with the reference genome as well as mutations published before 2009 were removed. After this processes, 258 LoF mutations and 129 GoF mutations remained. Second, with the remaining mutations, we extracted the features which have discriminative power between LoF and GoF mutations. Lastly, we implemented a classification process using the selected features to confirm whether they can be used to classify the two types. Here, we show six properties of mutations which can contribute to the identification of a LoF or a GoF outcome. These six are the subcellular location, the mutation subtype, the reference and substituted allele, the functional impact, and the protein domain.

## Method

### Text mining

Mutation information with the experimentally characterized LoF and GoF outcomes was collected from the literature. In this case, we extracted information about LoF and GoF outcomes from PubMed. First, we searched all PubMed abstracts which contain the acronyms "GOF" and "LOF" and the words "gain of function," "gain-of-function," "loss of function" and "loss-of-function" as keywords. Then, we found all related genes for each abstract and sentence containing the relevant genes using Gene2Pubmed [14]. Next, we tagged the mutations and their locations using tmVar, which is a previously published software, to extract mutation information from the literature : substitutions, insertions, deletions, and SNP and frameshift mutations of DNA and protein sequences, so we also used CRF features as mentioned in the reference [15].

### Data preprocessing

Once the LoF and GoF mutation information was collected from the literature, the mutation data set was preprocessed for further analysis. First, we selected LoF and GoF mutations that were published after the year 2010 in order to filter out mutations which had been identified against older versions of the reference genome. Second, mutations that are represented with an amino acid information were converted into those with a genomic location using the exon and intro information in Consensus CDS (CCDS, using GRCh37.p13) [16-18] so that we could observe the differences between LoF and GoF mutations at the nucleotide sequence level. Also, amino acid residues were converted into 3-mer nucleotide alleles by incorporating the CCDS nucleotide sequence and the amino acid codon table. In addition, substituted mutations were subgrouped into missense, nonsense and silent mutations according to the amino acid residue. During this step, silent mutations were removed. After these preprocessing steps, 258 LoF mutations and 129 GoF mutations remained.

### Mutation impact

In order to evaluate the significance of missense mutation effects on protein functions, the functional impact scores (FIS) of the LoF and GoF mutations were evaluated using the FIS method [13], which calculates the significance scores of point mutations based on evolutionary conservation of the mutation sites. We used the following information as the input of the FIS method: hg version, chromosome, mutation location, reference allele, substituted allele.

### Protein domain

To demonstrate the relationships between the protein domain functions and the LoF and GoF mutations, we used the Pfam database [19], which makes available large-scale protein domain information about proteins. We found protein domains corresponding to LoF and GoF mutation locations and formulated a distribution of the protein domains of the two classes with all 55931 human genes as the background. With this information, we performed a hypergeometric test to find the protein domains which had significantly different distributions.

### Machine learning

In order to classify LoF and GoF mutations based on our defined features, we utilized the support vector machine (SVM) with normalized poly kernel, random forest (RF) with 100 trees, logistic regression methods using the WEKA package [20,21]. Both SVM and logistic regression use linear decision boundaries, but unlike the logistic regression, not all instances affect building classifier function in the SVM. Only instances near the boundary are considered, and such difference between two classifiers can lead to different result. Six features were selected to build a classifier: the reference allele, the substituted allele, the mutation type, the mutation impact, the subcellular location and the protein domain. Among these features, the mutation impacts are continuous attributes, and the other features are nominal attributes. Then, WEKA package automatically transformed nominal attributes into binary ones if classifier cannot handle the nominal attributes. To avoid bias due to the different data sizes in the two classes, we randomly chose 100 instances of mutation data from each of the LoF and GoF data sets, after which the random selections were iterated 50 times, so 50 equal size data sets were made. For each data set, we performed five-fold cross validation and repeated the five-fold cross validation process 100 times. For each five-fold cross validation, 160 instances were used to train the classifiers, and 40 instances were used to test. As a result, we obtained 5,000 five-fold cross validation results. Table 1 shows an example of input instances.

**Table 1:** Input format example.

REF	SUB	TYPE	SCORE	SL	PD	Class
A	G	Missense	1.78	Cell membrane	Cadherin	GoF

T	G	Missense	3.62	Isoform2	SelR	LoF

T	A	Missense	-1.505	Apical cell membrane	ASC	LoF

Six features were used: the reference allele (REF), the substituted allele (SUB), the mutation type (TYPE), the mutation impact (SCORE), the subcellular location (SL) and the protein domain (PD).

## Results

### Tagged mutations from the literature

We obtained 14,259 gene-sentence relationships for GoF and 29,586 relationships for LoF. From these relationships, genes which did not have sentences were removed. We obtained 2,142 sentences for GoF and 4,600 sentences for LoF as a result. Next, tmVar [15] found 474 mutations for GoF and 816 mutations for LoF. Consequently, we obtained 474 mutations from 2,142 sentences for GoF and 816 mutations from 4,600 sentences for GoF.

### Overlapping genes

**Figure 2:**
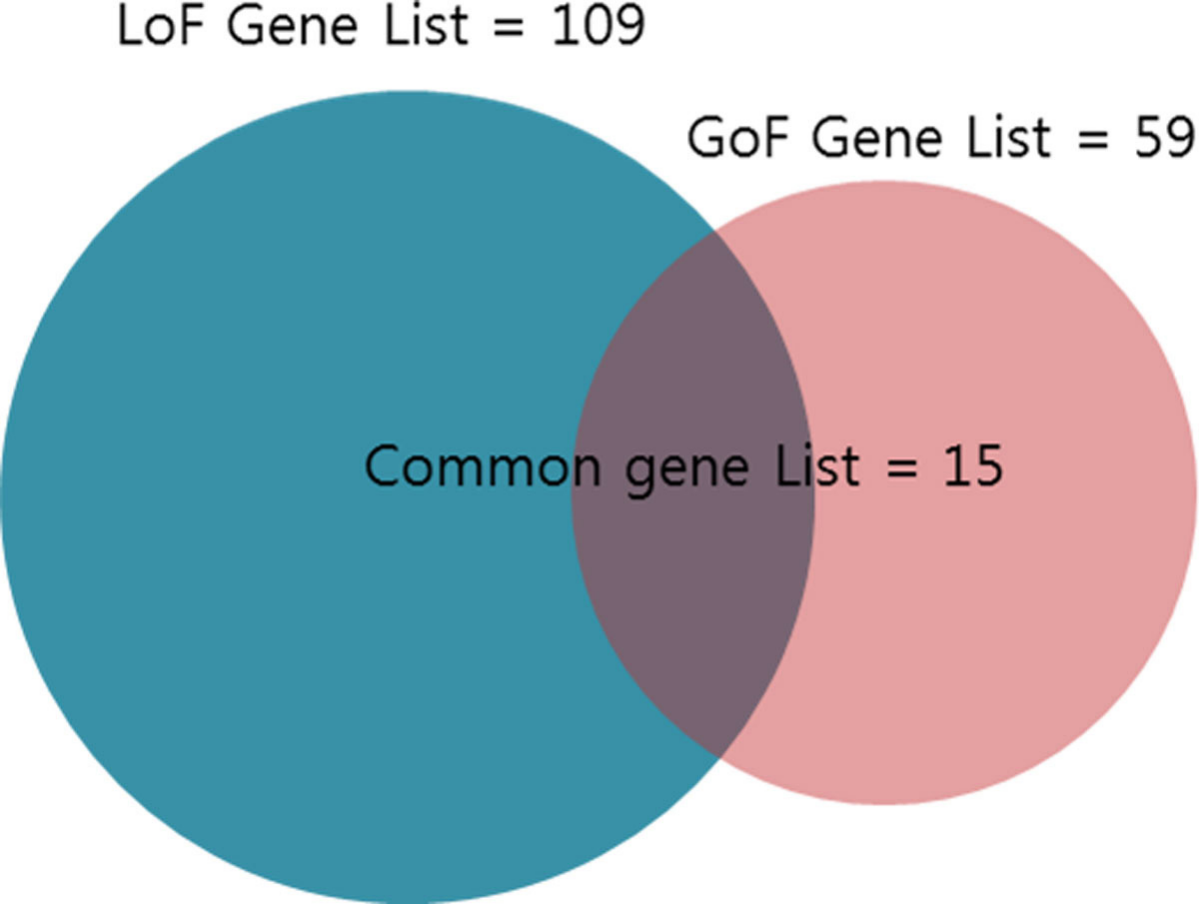
**The number of genes in LoF and GoF mutations**. The number of gene lists for LoF and GoF mutations

First, from the literature, we obtained mutation information for 816 LoF and 474 GoF mutations. Next, during the data-preprocessing step, mutations whose reference alleles were not matched with a reference genome or mutations published before 2009 were removed. As a result, there remained 258 LoF mutations and 129 GoF mutations. We extracted gene names from the 258 LoF mutations and 129 GoF mutations. Finally, 109 LoF genes and 59 GoF genes were selected for further analysis. Figure 2 demonstrates that there were 15 common genes. However, since the gene names are distributed broadly, there is no pattern that can be used to classify LoF and GoF mutations.

### Subcellular location

**Figure 3:**
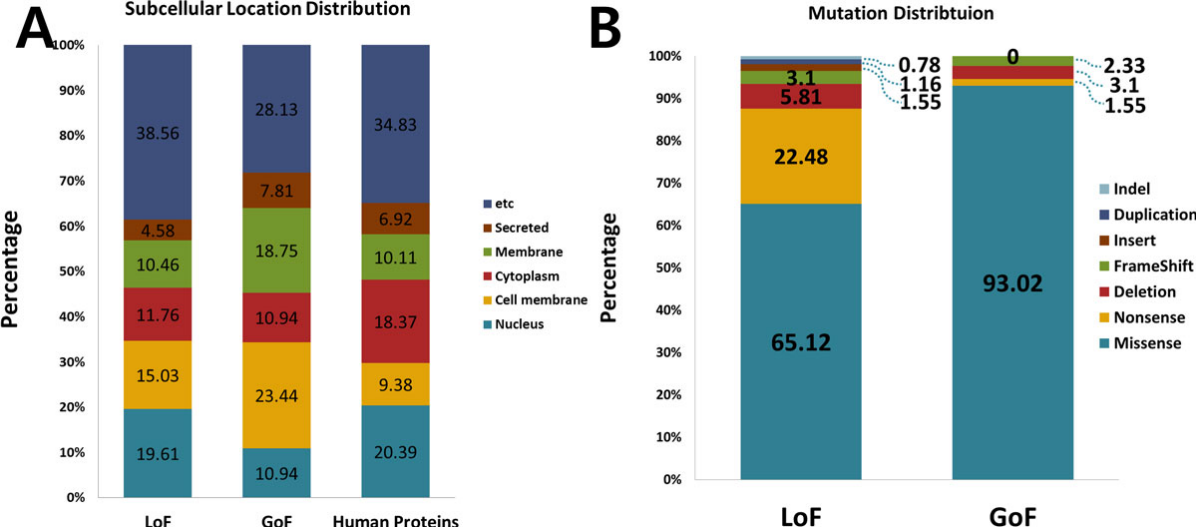
**Subcellular location distribution & mutation subtype distribution**. A. Distribution of the subcellular locations of LoF and GoF, and human mutations. The Y axis shows the percentage of each subcellular location in each class. B. Mutation compositions in the LoF and GoF, and human classes. The Y axis shows the percentage of each mutation type in each class.

The subcellular location information of the proteins was collected from the UniProt database [22]. We then analyzed the enriched subcellular locations of the LoF and GoF mutant genes using a hypergeometric test against the information of the total of 22119 subcellular locations of human proteins. Figure 3A shows the calculated distributions of the subcellular locations of the LoF and GoF genes, including in each case the nucleus, cell membrane, cytoplasm, membrane, and secreted. The subcellular location which contains the highest number of LoF mutated genes is the nucleus (19.61%), while in GoF it is the cell membrane (23.44%). When we implemented the hypergeometric test to compare the distributions between the LoF and the human results, and between the GoF and the human results, we found that the distributions of the subcellular locations of LoF mutations and the background subcellular locations were significantly different in the cell membrane and cytoplasm (P-value = 0.0159, 0.0180). In addition, the distributions of the subcellular locations of the GoF mutations and the background subcellular locations were significantly different in the nucleus, cell membrane, and membrane (P-value = 0.0356, 0.0001, 0.0254, respectively).

### Mutation subtypes

Next, we extracted mutation subtypes from the LoF and GoF mutations and compared their distributions. In this work, we used six types of mutations: missense, nonsense, deletion, indel, duplication and frame shift. Figure 3B shows the distribution of the mutation subtypes of LoF and GoF. MacArthur *et al*. studied LoF variants, but did not focus on the missense mutations [4]. However, our study shows that the most frequently found type of mutation is the missense mutation in both cases for LoF and GoF mutations. This ratio indicates that missense mutations are also an important proportion of the mutations which affect protein functions. The second most frequently found mutation is the nonsense mutation in LoF; for GoF, it was the deletion mutation. These results indicate that nonsense mutations usually lead to a protein which causes a loss of function and not a gain of a new function.

### Reference and substituted allele ratio

**Figure 4:**
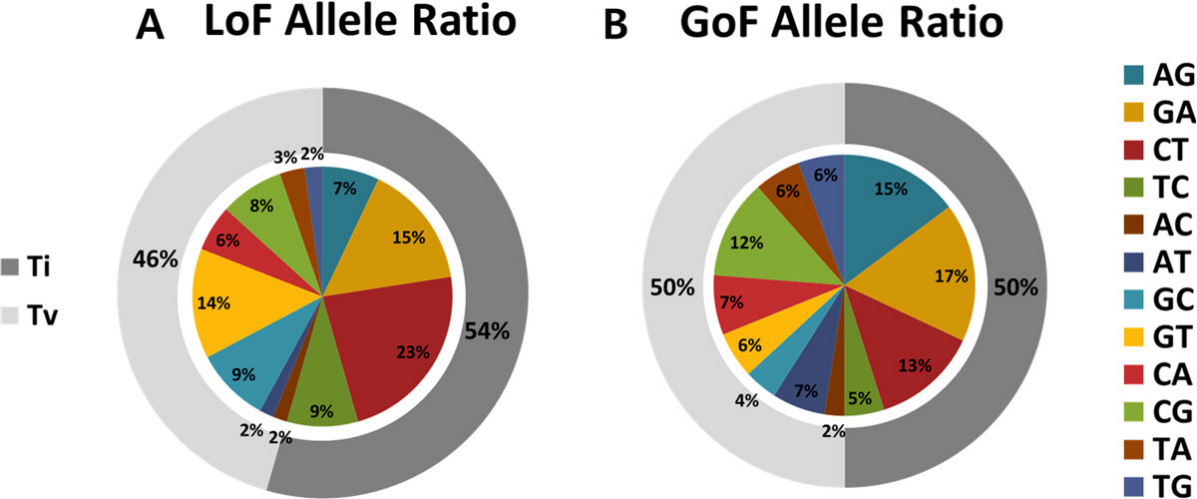
**Allele**. A. Reference to substituted allele rates and transition and transversion (Ti, Tv) rates in LoF. B. Reference to substituted allele rates and transition and transversion (Ti, Tv) rates in GoF.

We extracted the nucleotide reference alleles and substituted alleles and classified allele pairs into two groups based on their nitrogenous bases. If a reference allele and a substituted allele were of the same nitrogenous base (purine and purine, pyrimidine and pyrimidine), the mutation was classified as a transition (Ti). Otherwise, if the two nitrogenous bases were different, it was classified as a transversion (Tv). We then analyzed the differences in the proportions of the each allele pairs between the LoF and GoF mutations. Figure 4 shows the ratio of reference and the substituted allele pairs, and Table 2 describes P-values pertaining to the result of comparing the LoF and GoF mutations using the propositional binomial. There were no significant differences found in the TiTv ratio between LoF and GoF, but for the LoF case, the transition (Ti) percentage is higher than the transversion (Tv) percentage. In addition, the AG, CT, AT, and GT results show significant differences (P-values: 0.0347, 0.0376, 0.0426, and 0.0399, respectively).

**Table 2:** Allele P-value.

Allele Pair	P-value
AG	0.0347

GA	0.7908

CT	0.0376

TC	0.2639

Transition	0.4987

AC	0.9707

AT	0.0426

GC	0.1224

GT	0.036

CA	0.7162

CG	0.261

TA	0.3627

TG	0.158

Transversion	0.4987

P-values from the comparison of the proportions of 12 references, i.e., the substituted allele pairs and the transition (Ti) and transversion (Tv) ratios between the LoF and GoF mutations.

### Protein domain

**Figure 5:**
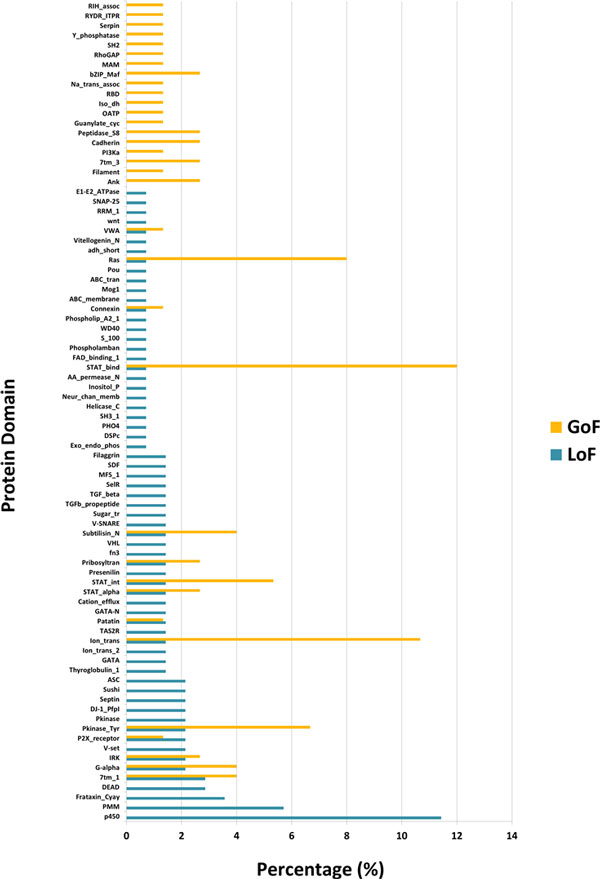
**Protein Domain**. LoF and GoF rates in protein domains

We collected the protein domain function information of each mutation. Figure 5 shows the distribution of the protein domain functions of the LoF and GoF mutations. The protein domain functions are distributed broadly, but some of them show several exclusive cases between the two classes. This result indicates that LoF and GoF mutations tend to affect different protein functions. Next, we analyzed the protein domain functions of the LoF and GoF mutations using a hypergeometric test against 55931 human protein domain functions.

### Mutation impact

**Figure 6:**
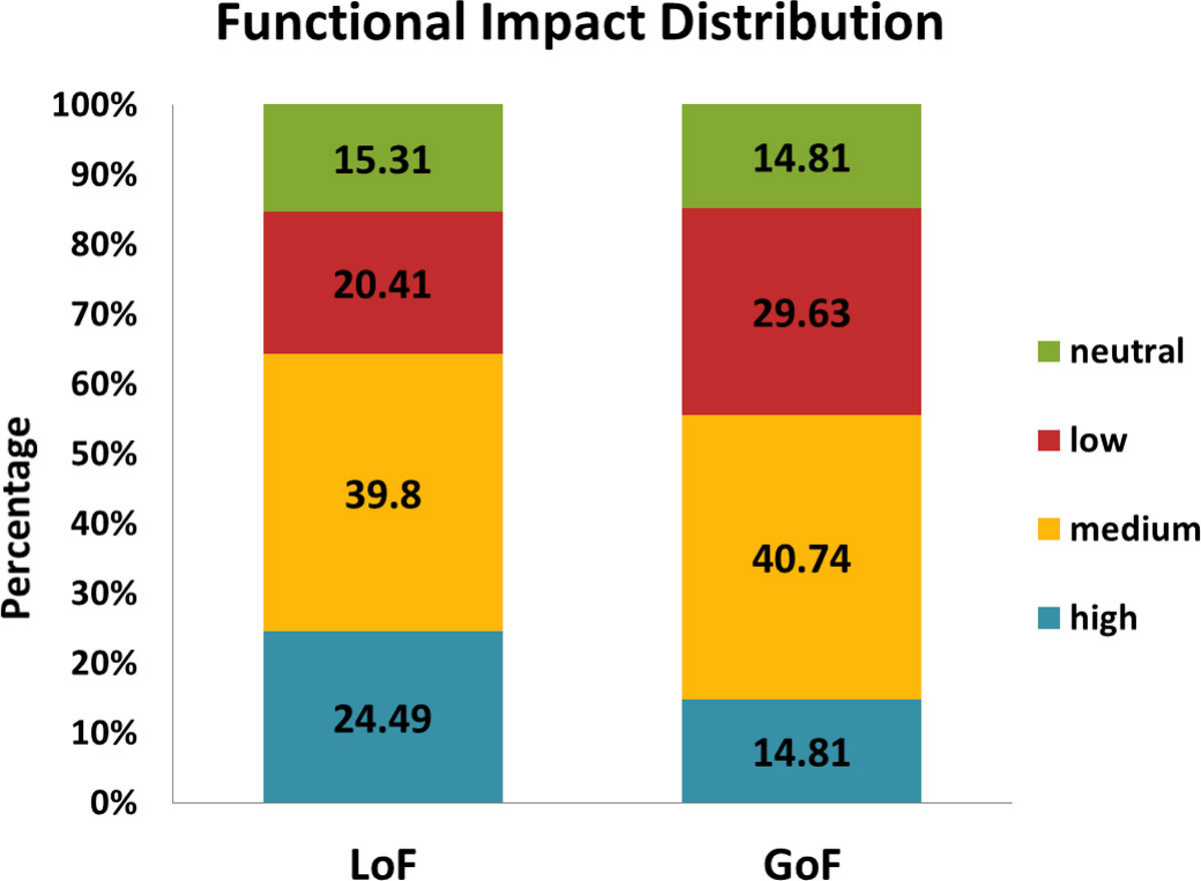
**Mutation Impact**. Distribution of the functional impact of missense mutations. The functional impact is the estimation how much the mutation affects the protein function. The Y axis shows the percentage of each functional impact in each class.

The FIS method was used to estimate the significance of the missense mutation effects on the protein functions, classifying mutations into four grades based on the estimated scores: high, medium, low, and neutral [13]. Mutations classified as a higher grade mutation have a more of an effect on protein functions than those classified as a lower grade mutation. Figure 6 shows the distributions of the functional impact grades of the LoF and GoF mutations. The percentages differ in high-impact mutations as compared to low-impact mutations. The LoF results shows a higher percentage of high-impact mutations than the GoF results (LoF: 24.49%, GoF: 14.81%) as well as a lower percentage in low-impact mutations (LoF: 20.41%, GoF: 29.63%). This result indicates that LoF mutations affect the protein function more than GoF mutations.

### Classification of LoF versus GoF with selected features

**Figure 7:**
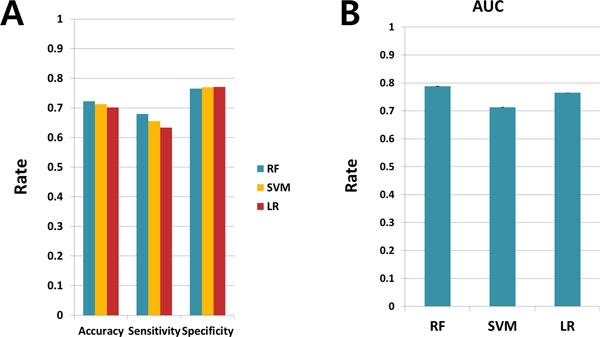
**Classification Result**. A. Accuracy, sensitivity, and specificity rates of the three classifiers used here: linear logistic regression, random forest (RF), and support vector machine (SVM). B. AUC values of the three classifiers.

To confirm whether or not the properties can be used as criteria for distinguishing LoF and GoF mutations, we implemented a classification technique using the support vector machine, random forest, and linear logistic regression methods with 50 data sets which contain equal numbers of LoF and GoF mutations to avoid bias. As a result of five-fold cross validation repeated 100 times, we obtained 25,000 results for each classifier and calculated the averages of the total accuracy, true positive rates (rates of LoF correctly classified as LoF), and true negative rates (rates of GoF correctly classified as GoF). Figure 7A shows the accuracy rates, the true positive rates (sensitivity), and the true negative rates (specificity); while Figure 7B shows the AUC rates. The average percent correctly classified was 71.28% for the support vector machine method, 72.23% for the random forest method, and 70.19% for the linear logistic regression method, while the AUC values for each classification were 0.7128, 0.7880, and 0.7646. From these results, we can confirm the discriminative power of the six features.

## Conclusion and Discussion

By mining the literature, 14,259 gene-sentence relationships for GoF and 29,586 relationships for LoF were collected. From these, genes without sentences were removed. Thus, 2,142 sentences for GoF and 4,600 sentences for LoF remained. We then tagged the mutations and their locations from the sentences. Consequently we obtained 474 mutations from 2,142 sentences for GoF and 816 mutations from 4,600 sentences for GoF. In addition, during the data-preprocessing step, mutations whose reference allele was not matched with a reference genome or mutations published before 2009 were removed. Hence, we analyzed 258 LoF mutations and 129 GoF mutations. As a result, we found six features which can distinguish LoF and GoF mutations: the subcellular location, the mutation subtype, the reference and substituted allele, the functional impact, and the protein domain. We used these features for classification to confirm whether or not they can identify LoF and GoF mutations. Finally, we obtained 72.23% accuracy for the random forest, 71.28% accuracy for the support vector machine, and 70.19% accuracy for the linear logistic regression methods, with AUC values of 0.7880, 0.7128, and 0.7646, respectively. As a result, we can conclude that the selected features can contribute to the identification of LoF and GoF mutations.

## Limitations and Future Work

Since the LoF and GoF mutation data were derived from the literature, the number of mutation data was limited and was not enough to understand overall tendency of the LoF and GoF mutations. In addition, although we selected mutations that were published after the year 2010, there were mutations not matched with the reference genome. In this work, we studied the LoF and GoF mutation properties, and we expect that this study can contribute to better understanding of the mutation effects on the biological systems. Through the analysis of associations between mutations and protein functions and the analysis of how the affected proteins influence the biological pathways, we can clarify biological mechanism from mutations to systems.

## Competing interests

The authors declare that they have no competing interests.

## Authors' contributions

SJ conducted the analysis and drafted the manuscript. HN and SK designed and coordinated the study. SL performed the literature data mining.

## Declaration

Publication costs for the article were sourced from GIST College's 2014 GUP Research Fund and by the "Systems Biology Infrastructure Establishment Grant" provided by the Gwangju Institute of Science & Technology in 2014.

This article has been published as part of *BMC Medical Informatics and Decision Making *Volume 15 Supplement 1, 2015: Proceedings of the ACM Eighth International Workshop on Data and Text Mining in Biomedical Informatics. The full contents of the supplement are available online at http://www.biomedcentral.com/bmcmedinformdecismak/supplements/15/S1.
